# A portable infrared photoplethysmograph: heartbeat of *Mytilus galloprovincialis* analyzed by MRI and application to *Bathymodiolus septemdierum*

**DOI:** 10.1242/bio.020909

**Published:** 2016-10-19

**Authors:** Eriko Seo, Toshiyuki Sazi, Morio Togawa, Osamu Nagata, Masataka Murakami, Shigeaki Kojima, Yoshiteru Seo

**Affiliations:** 1 Department of Marine Ecosystem Dynamics, Division of Marine Life Science, Atmosphere and Ocean Research Institute, The University of Tokyo, Kashiwa 277-8564, Japan; 2 Department of Molecular Physiology, National Institute for Physiological Sciences, Okazaki 444-8787, Japan; 3 Department of Regulatory Physiology, Dokkyo Medical University School of Medicine, Tochigi 321-0293, Japan

**Keywords:** Cardiac cycle, Cardiac arrest, Arrhythmia, Bivalve, Plethysmography, MRI

## Abstract

Infrared photoplethysmogram (IR-PPG) and magnetic resonance image (MRI) of the *Mytilus galloprovincialis* heart were obtained simultaneously. Heart rate was varied by changing temperature, aerial exposure and hypoxia. Higher heart rates (35-20 beat min^−1^) were usually observed at 20°C under the aerobic condition, and typical IR-PPG represented a single peak (peak *v*). The upward and downward slopes of the peak *v* corresponded to the filling and contracting of the ventricle, respectively. A double-peak IR-PPG was observed in a wide range of heart rates (5 to 35 beats min^−1^) under various conditions. The initial peak *v* corresponded to the filling of the ventricle, and the origin of the second peak (*v’*) varied with the heart rate. A flat IR-PPG with a noise-level represented cardiac arrest. Although large movement of the shells and the foot caused slow waves or a baseline drift of the IR-PPG, the heart rate can be calculated from the *v-v* interval. Based on these results, we assembled a portable IR-PPG recording system, and measured the heartbeats of *Bathymodiolus septemdierum* (Mytilidae) for 24 h on a research vessel just after sampling from the deep sea, showing that IR-PPG is a noninvasive, economical, robust method that can be used in field experiments.

## Abstract (precis)

**Summary:** Infrared photoplethysmogram of *Mytilus* heart was analyzed by magnetic resonance imaging. Portable photoplethysmographs provide a noninvasive, economical and robust method to monitor the heartbeat of mussels in field experiments.

## INTRODUCTION

The heartbeat is one of the four vital signs that reflect a body in a homeostatic balance (Carroll, 2007). Changes in cardiac activity are considered as integrative measures of the physiological fitness of organisms (Hagger et al., 2008). Indeed, the physiology and pharmacology of the hearts of bivalve molluscs have been studied for many years (Bayne, 1976), investigating a variety of important factors, such as the effects of temperature (Zittier et al., 2015; Bayne et al., 1976), salinity (Sara and De Pirro, 2011), aerial exposure (Coleman and Trueman, 1971), CO_2_ tension (Zittier et al., 2015) and exposure to heavy metal ions (Curtis et al., 2000; Bakhmet et al., 2012). By measuring the heartbeat in field experiments and outside of the laboratory, we may be able to clarify the vivid activity of bivalves, and furthermore, minimize the delay of starting the heartbeat measurements after sampling of bivalves.

Two invasive techniques have been used for measuring the heartbeat in bivalves: (a) direct observation of the heartbeat through a small window cut in the shell, and (b) observation of changes in impedance using a pair of small electrodes implanted along the side of the pericardium (Bayne, 1976). Laser Doppler and ultrasound imaging are essentially noninvasive techniques, and have been applied in studies of the heart of zebrafish (Sun et al., 2008). However, in bivalves, due to a thick shell wall we have to make a small hole to insert the laser probe near the heart (Lannig et al., 2008). Infrared photoplethysmography (Bakhmet et al., 2015; Curtis et al., 2000; Depledge and Andersen, 1990) has advantages for application in field experiments, as it is possible to conduct (a) real-time monitoring and (b) day-long continuous measurements non-invasively and also (c) because it requires only low-cost hardware (Depledge and Andersen, 1990); however, until this study, the relationship between the results obtained by infrared photoplethysmogram (IR-PPG) and the actual heartbeat has not been confirmed in bivalves. This is a major reason why usage of the IR-PPG is limited. In this study, the cardiac cycle of the *Mytilus galloprovincialis* was observed by infrared photoplethysmograph (IR-PP) and T_1_-weighted gradient-echo magnetic resonance image (T_1w_- MRI) simultaneously. Then, the waveform of the IR-PPG was analyzed by comparing it with the cardiac cycle determined by T_1w_-MRI (Seo et al., 2014a,b). In addition, we assembled a portable IR-PPG recording system, and tried to measure the heartbeat of a deep-sea bivalve, *Bathymodiolus septemdierum* (Mytilidae), on a research vessel just after sampling from the deep sea.

## RESULTS AND DISCUSSION

### IR-PPG during regular heartbeat

**Fig. 1.**
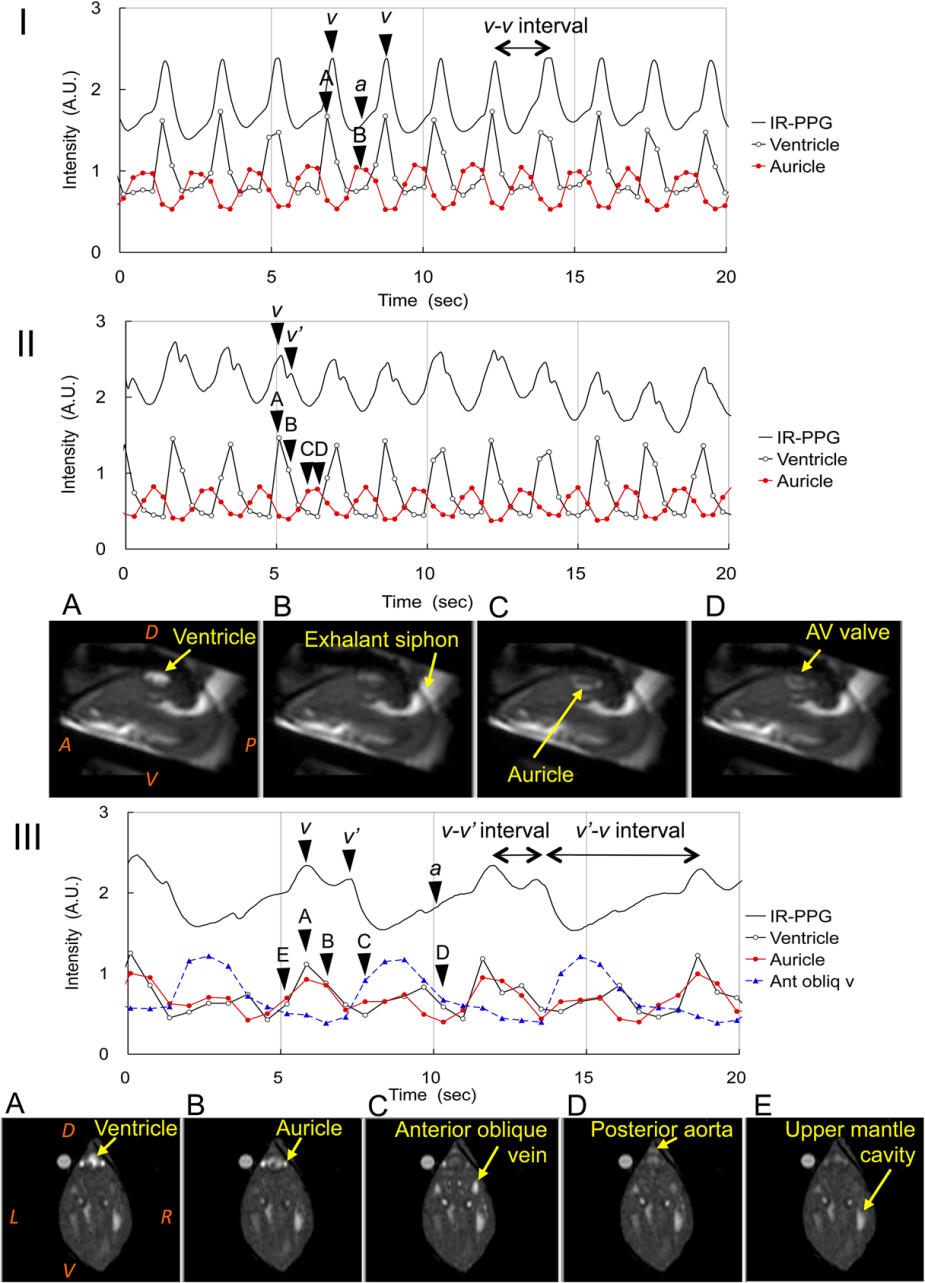
**IR-PPG and T_1w_-MRI of regular heartbeat of *Mytilus galloprovincialis.*** The IR-PPG is shown as a solid line. The T_1w_-MR image intensities of the ventricle, auricle and anterior oblique vein are shown as open circle, closed circles and triangles, respectively. (I) Single-peak IR-PPG observed at 21°C in the immersed condition. The labels *v* and *a* indicate a sharp peak and a slow increase, respectively. The timing of the sagittal T_1w_-MRI is shown by the arrowheads labeled A to B. A, ventricular filling and contraction of the hemolymph into the ventricle. B, filling of the hemolymph into the auricles. The heart rate calculated from the *v-v* interval and that from the peak-peak interval of the ventricle were 32.4±1.5 bpm (*n*=43) and 32.5±3.2 bpm (*n*=43), respectively. The number (n) represents the number of heartbeats used for the calculations. (II) IR-PPG of a single peak with a small splitting observed at 21°C in the immersed condition*.* The labels *v* and *v’* indicate a main peak and a small second-peak, respectively. The timing of the sagittal T_1w_-MRI is shown by the arrowheads labeled A to D, and detailed in the corresponding panels A-D underneath. A, end of the filling of hemolymph into the ventricle. B, ventricular contraction. C, filling of the hemolymph into the auricles. D, opening of the AV valve. The heart rates calculated from IR-PPG and MRI were 34.4±0.81 bpm (*n*=22) and 34.9±3.1 bpm (*n*=22), respectively. (III) Double-peak IR-PPG observed at 10°C in the emersed hypoxic condition. The labels *v, v’* and *a* indicate two peaks and a slow increase, respectively. The timing of the transverse T_1w_-MRI is shown by the arrowheads labeled A to E, and detailed in the corresponding panels A-E underneath. A, end of the filling of hemolymph into the ventricle. B, start of the ventricular contraction. C, filling of the hemolymph into the anterior oblique vein. D, end of ventricular contraction. Flows in the ventricle and posterior aorta still continued. E, opening of the AV valve. The heart rates calculated from IR-PPG and MRI were 9.37±0.67 bpm (*n*=11) and 9.35±0.78 bpm (*n*=11), respectively. In panels IIA and IIIA, A, anterior; P, posterior; D, dorsal; V, ventral; R, right; L, left.

Previously, Depledge proposed measuring the heart rate of the crab by using IR-PP (Depledge, 1984). Compared with direct observation of the heartbeat via a small hole drilled in the carapace, it has been confirmed that reflection of IR light decreased during the systolic phase, while reflection of the IR light increased during the diastolic phase (Depledge and Andersen, 1990). However, the IR-PPG waveform of *Mytilus galloprovincialis* varied from a single peak to a double peak, and sometimes there was a split into more than three peaks (Figs 1 and 3).

The heartbeats of 11 mussels were measured under various conditions, such as immersed, emersed, and at rapid exposure of lower temperature. Among the 139 measurement sessions, a normal regular rhythm was observed in 64.7% of the sessions. At temperatures around 20°C, higher heart rates (35-20 bpm) were observed, and the IR-PPG usually showed a single peak consisting of a slow increase (*a*) and a sharp peak (*v*) (Fig. 1I). Compared with sagittal T_1w_-MRI, the upward slope of the peak *v* coincided with the filling of the hemolymph into the ventricle, and the downward slope of the peak *v* coincided with the contraction of the ventricle (Fig. 1IA). The slow increase *a* coincided with the filling of the hemolymph into the auricles (Fig. 1IB).

Double peak IR-PPGs were observed in a wide range of heart rates, from 35 bpm to 5 bpm. Even at a high heart rate (around 35 bpm), the peak of the IR-PPG often split into two peaks (Fig. 1II). Compared with sagittal T_1w_-MRI (Fig. 1II; Movie 1), the upward slope of the peak started from the opening of the auriculoventricular (AV) valve (Fig. 1IID), and the peak *v* coincided with the end of the filling of the hemolymph into the ventricle (Fig. 1IIA). The small peak (*v’*) coincided with the ventricular contraction (Fig. 1IIB). In this case, the filling of the hemolymph into the auricles (Fig. 1IIC) coincided with the minimum of the IR-PPG waveform.

The intermediate heart rates (10-20 bpm) were usually observed during a decrease in temperature to 10°C, as reported by Zittier et al. (2015). The IR-PPG consisted of two peaks (*v*, *v’*) and a slow increase (*a*) (Fig. 1III). Compared with transverse T_1w_-MRI (Fig. 1III; Movie 2), the upward slope of the peak *v* started from the opening of the AV valve (Fig. 1IIIE), and the peak *v* coincided with the end of the filling of the hemolymph into the ventricle and auricles (Fig. 1IIIA). The second peak (*v’*) was delayed, appearing at the start of ventricular contraction (Fig. 1IIIB). The systolic period continued until just before the opening of the AV valve (Fig. 1IIID). During the systolic period not only the flow in the posterior aorta, but also the flow in the anterior oblique veins, increased significantly and the later coincided with the slow increase *a* of the IR-PPG waveform.

At an even slower heart rate (5 bpm), usually observed at 10°C in the emersed hypoxic condition, the separation between the peaks *v* and *v’* became significant, so that the *v-v’* interval became similar to the *v'-v* interval (data not shown). Compared with the sagittal T_1w_-MRI, the upward slope of the peak *v* started from the opening of the AV valve, and the peak coincided with the filling of the hemolymph into the ventricle. The second peak (*v’*) coincided with the filling of the hemolymph into the auricles.

**Fig. 2.**
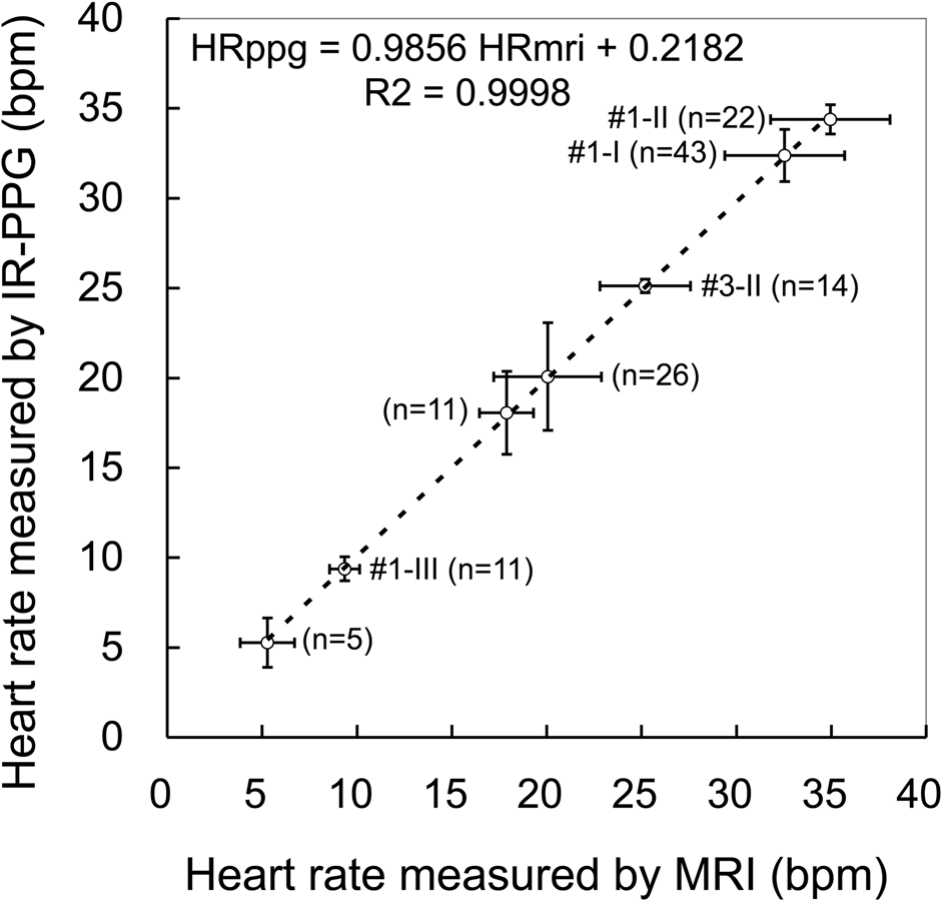
**Heart rate observed by MRI and IR-PPG*.*** Correlation of the heart rate calculated from the *v-v* interval of the IR-PPG (HRppg) and that from the interval of ventricular contraction detected by MRI (HRmri). Means and s.d. of seven sessions from four mussels are shown. Data labeled #1 and #3 were obtained in the sessions shown in Fig. 1 and Fig. 3, respectively. The number (n) represents the number of heartbeats used for the calculations. R^2^ is a coefficient of determination of the regression line estimated from the seven sessions.

From these results, the initial peak *v* corresponded to the filling of the ventricle, and the origin of the second peak (*v’*) varied with the heart rate. Therefore, the heart rates can be calculated from the *v-v* interval (Fig. 1I). Indeed, compared with the heart rates calculated from the *v-v* interval and that from the interval of the ventricular contraction detected by MRI, the slope approaches unity, with a high correlation coefficient (Fig. 2).

**Fig. 3.**
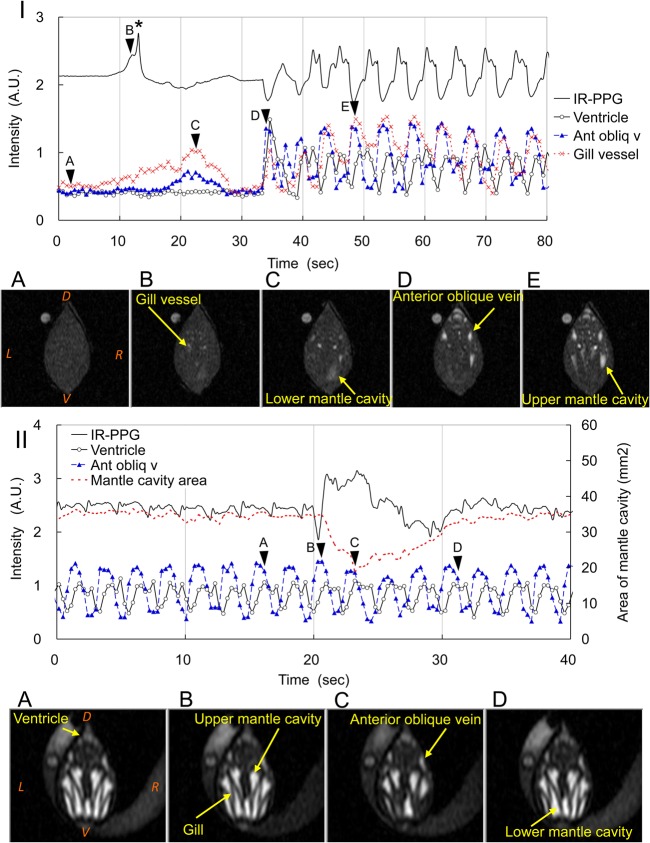
**Artifacts in IR-PPG of *Mytilus galloprovincialis.*** The IR-PPG is shown as a solid line. The area of the water flow in the mantle cavity is shown as a dotted line. The T_1w_-MR image intensities of the ventricle, anterior oblique vein and gill vessel are shown as open circle, triangles, and crosses, respectively. (I) Restart of the heartbeat after cardiac arrest observed at 20°C in the emersed condition. The timing of the transverse T_1w_-MRI is shown by the arrowheads labeled A to E, and detailed in the corresponding panels A-E underneath. A, cardiac arrest. B, slow increase of flow in the gill vessels. C, slow increase of flows in the anterior oblique veins and the lower mantle cavity. D, ventricular contraction restarted. E, ventricular contraction three beats after the start of the heartbeat. Asterisk indicates peak of an unknown origin. (II) Slow wave in IR-PPG and close/open process of the shells observed at 20°C in the immersed condition. The timing of the transverse T_1w_-MRI at the end of the filling of the ventricle is shown by the arrowheads labeled A to D, and detailed in the corresponding panels A-D underneath. A, the mussels kept their shells open. B, the mussels started to closure their shells. C, the mussels closed their shells. D, the mussels reopened their shells. The upper and lower mantle cavities are divided by the W-shaped gill depicted black in T_1w_-MRI. In panels IA and IIA, D, dorsal; V, ventral; R, right; L, left.

### Arrhythmia, cardiac arrest and restart of the heartbeat

Arrhythmia could be detected by change of the *v-v* interval. Among the 139 measurements sessions, arrhythmia appeared in 16.5% of the sessions. The results obtained by MRI and IR-PPG agreed well with each other (data are not shown). Mussels spontaneously arrested or restarted heartbeat during 18.7% of the sessions. A typical case of a restart of the heartbeat after cardiac arrest (2.5 min), observed at 20°C in the emersed hypoxic condition, is shown in Fig. 3I and Movie 3. During cardiac arrest, the IR-PPG was almost flat, and we did not detect any cardiac motion in the transverse T_1w_-MRI (Fig. 3IA); therefore, a flat IR-PPG with a noise-level represented cardiac arrest. The mussel restarted the heart almost instantaneously at around 35 s (Fig. 3I). The ventricular contraction and the auricular filling appeared simultaneously, and caused a sharp drop in the IRP waveform (Fig. 3ID). After the fourth beat, the IR-PPG waveform seemed to be steady with a constant *v-v* interval.

### Artifacts in the IR-PPG

On occasion, peaks, slow waves or a drift in the baseline appeared in the IR-PPG, and these are not likely related to the heartbeat. A typical result observed at 20°C under immersed condition is shown in Fig. 3II and Movie 4. A slow wave started when the mussel started to close the shells (Fig. 3IIB), and the amplitude became maximum when the shells closed (Fig. 3IIC). Then, the wave decreased to the initial level when the mussels opened their shells again (Fig. 3IID). During this period, the heart rate measured by T_1w_-MRI was almost a constant (23.6±2.4 bpm, *n*=4), and it was similar to that for the whole of the session (25.1±2.9 bpm, *n*=33). In separate experiments, peaks were detected when the internal organ moved to the ventral side (data are not shown). When the heartbeats were regular, the beating of the heart could be detected by IR-PPG. Peaks, slow waves or a drift in the baseline were caused by movement of organs, such as the foot, or by opening/closing of the shells; however, we could still detect the peak *v* and calculate the heart rate.

Typical artifacts during cardiac arrest are shown in Fig. 3I. In this mussel, at around 25 s before the restart, a broad and a sharp peak appeared in the IR-PPG. However, there was little change in the T_1w_-MRI, except for an increase of flow in the gill vessels (Fig. 3IB). An increase in the flow in vessels and the lower mantle cavity had a minimum effect on the IR-PPG (Fig. 3IC). Therefore, broad peaks, slow waves or a drift in the baseline that appeared in the IR-PPG could be assigned as artifacts. However, the sharp peaks shown during cardiac arrest (indicated by an asterisk in Fig. 3I) made it difficult to discriminate between the peaks *v* and artifacts. This is a limitation of the IR-PPG method for measuring the heartbeat, and this limitation illustrated the need for T_1w_-MRI to judge whether a peak represented the heartbeat or an artifact.

It would be fair to mention that the intensity of an IR-PPG depends not only on volume, but also on the characteristics of the IR sensor and the IR-PP circuit (Fig. S1). Therefore, we have to be more careful when reading an IR-PPG obtained at a lower heart rate. For example, the drop between the peak *v* and *v*’, and also the slow increase *a* in Fig. 1III, might be enhanced by the characteristics of the IR sensor and the IR-PP circuit. In regard to detection of the peak *v*, this IR-PP hardware works over a wide range of heart rates.

### Measurement of the heartbeat of *Bathymodiolus septemdierum* on a research vessel

**Fig. 4.**
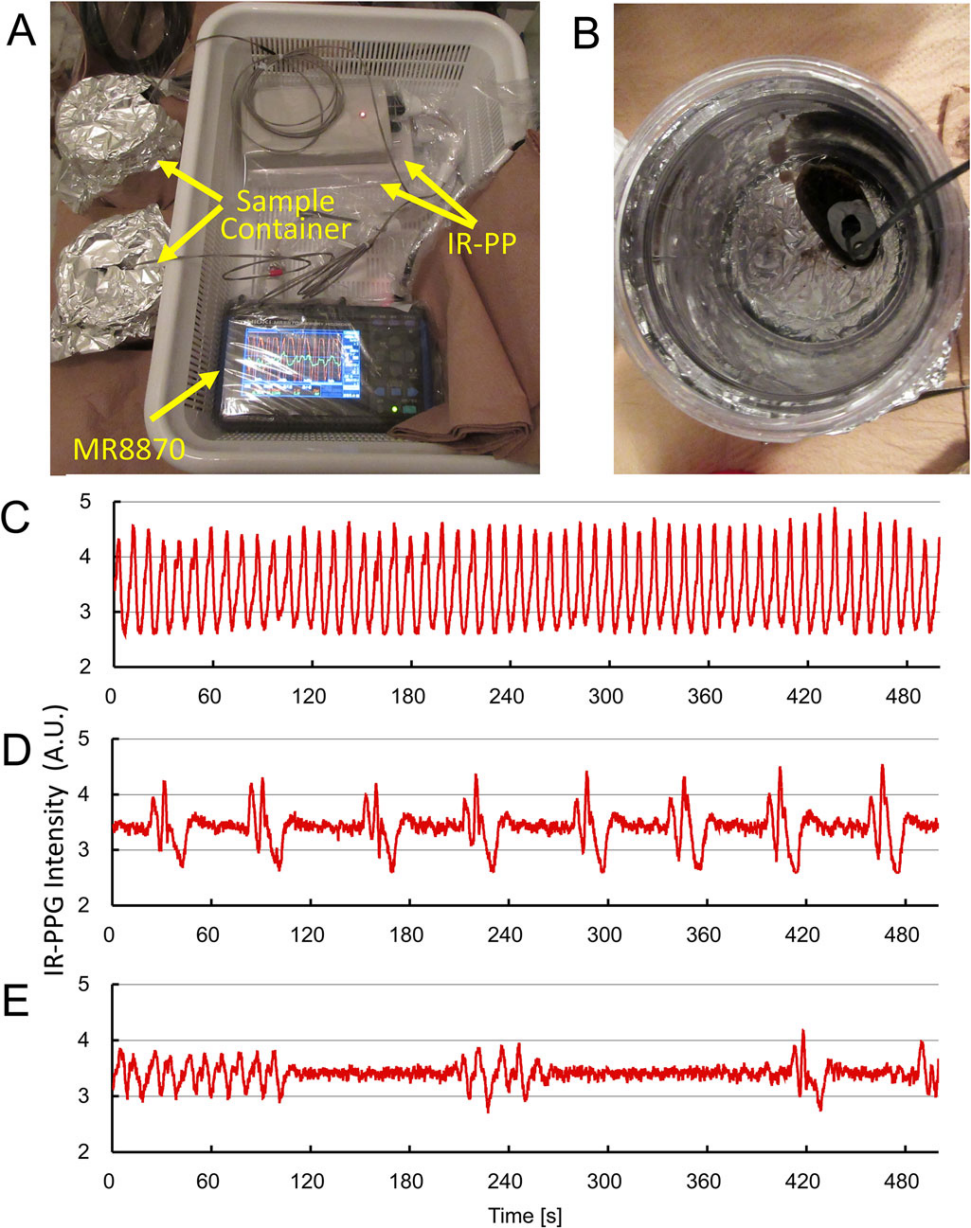
**Portable IR-PPG recording system and IR-PPG of *Bathymodiolus septemdierum* at 5°C.** (A) The IR-PPG recording system for the cold room in the research vessel, *Kaiyo*. It consists of two boxes of IR-PP, an MR8870 digital data recorder, and two IR sensors in sample containers. Total weight of the system is around 1.5 kg, and it can set in a space of 40 cm square. (B) *Bathymodiolus septemdierum* and an IR sensor fixed with adhesive rubber. (C-E) IR-PPG of *Bathymodiolus septemdierum* at 5°C recorded in the research vessel, *Kaiyo*, within 24 h after sampling. (C) Regular heartbeat observed at 23 h 40 min after sampling. The heart rates calculated from IR-PPG were 6.53±0.25 bpm (*n*=54). (D) Bradycardia observed at 21 h 20 min after sampling. The heart rates calculated from IR-PPG were 0.97±0.07 bpm (*n*=7). (E) Arrhythmia including cardiac arrest observed at 20 h 00 min after sampling. The number (n) represents the number of heartbeats used for the calculations.

From the basis of results shown in the above, a combination of IR-PPG and *T*_1w_-MRI allowed us to analyze IR-PPG waveforms in a wide range of heart rates in *Mytilus*, and it was possible to calculate the heart rate from the *v-v* interval. The origin of artifacts shown on the IR-PPG could be determined by using T_1w_-MRI; therefore, we tried to assemble a portable IR-PPG recording system for measuring heartbeat of deep sea bivalve on a research vessel. The system should be a compact and able to operate at 5°C, and capable of recording IR-PPG continuously for more than 24 h. The IR-PP circuit reported by Burnett (Burnett et al., 2013) is compact and has low energy consumption; the length, width, thickness and weight of the this IR-PP are 170 mm, 80 mm, 35 mm and 300 g including four AA batteries (LR6) (Fig. 4A), and it works more than 48 h continuously at 5°C. An MR8870 digital data recorder was used for IR-PPG recording, with a length, width, thickness and weight of 176 mm, 101 mm, 41 mm and 600 g, respectively, including a rechargeable battery (Fig. 4A). The MR8870 works at a range of temperatures from 0 to 40°C, and it has a data size of 52 kB for a recording of two channels of IR-PPG for 500 s with a sampling interval of 50 ms; it is possible to store more than 1 month of data in a compact flash memory of MR8870 (1 GB). As shown in Fig. 4A,B, the IR-PPG recording system for the cold room in the research vessel, *Kaiyo*, needed only 40 cm square of space. In addition, the internal battery of MR8870 could work for more than 1 h, therefore, it is possible to measure IR-PPG without an AC line when the sample was transported from the vessel to a laboratory on shore.

Recording of the heartbeats of two *Bathymodiolus* mussels were started 4 h after sampling, and measured for 24 h. Even with the sway and vibration of the vessel, we could detect fairly stable IR-PPG. A regular heartbeat at around 7 bpm (Fig. 4C), a slow heartbeats (bradycardia) (Fig. 4D), and arrhythmia including cardiac arrest (Fig. 4E) were observed, and these seemed to be appeared at random. It is also likely that variation of heartbeats was larger than that observed at 2 weeks after the sampling (data are not shown). Bradycardia and cardiac arrest were reported in bivalves, such as *Mytilus*, *Isognomum* and *Anodonta* (Curtis et al., 2000; Seo et al., 2014a; Trueman and Lowe, 1971; Brand, 1976). The decrease of heart rate was caused by not only the metabolic suppression by hypoxia, but also was linked to various activities of bivalves such as closure of shells and extension of the pedal (de Zwaan and Wijsman, 1976; Curtis et al., 2000; Brand, 1976). Therefore, like patients of arrhythmia, continuous monitoring of the heart rate must be useful to find factors responsible to control the heartbeat.

From results obtained by this pilot experiment, the portable IR-PPG recording system is cheap, simple and robust and can be used in field experiments. In future, we will develop an IR-PP for *in situ* experiment in the deep sea.

## MATERIALS AND METHODS

### Experimental animals

*Mytilus galloprovincialis* Lamarck 1819 were supplied by Fishing Ito Limited (Chita, Aichi, Japan). These mussels were collected from a tidal area along the shore of Nagoya harbor, on July 2015. At the laboratory, in two separate 5 liter baths, 10 mussels were kept in each bath for a week in aerated synthetic seawater (salinity 3.6%) at room temperature (20-24°C) (Seo et al., 2014b). A total of 11 mussels were used in this MRI study. The length, height and width of the mussels were 27.4±0.43 mm, 14.6±0.34 mm, 8.7±0.27 mm (means and s.e.m.), respectively.

*Bathymodiolus septemdierum* Hashimoto and Okutani 1994, a family of Mytilidae, were collected at a depth of 1182 m at a seep site around Myojin Knoll in the Northern Ogasawara Islands (32° 7.48′N, 139° 50.534′ E) during the KY15-07 cruise (27 April 2015), using the remotely operated vehicle (ROV) *Hyper-Dolphin* (Dive HPD#1810), operated by the research vessel *Kaiyo* of the Japan Agency of Marine-Earth Science and Technology (JAMSTEC). Two of these mussels were used in this MRI study. The length, height and width of the mussels were 46.5 and 46.2 mm, 28.6 and 28.4 mm, 18.0 and 18.4 mm, respectively.

All of the animal experiments conducted in this study were carried out under the rules and regulations of the ‘Guiding Principles for the Care and Use of Animals’ set by the Physiological Society of Japan, and approved by the Animal Research Councils at Dokkyo University School of Medicine (#840).

### Infrared photoplethysmography

An infrared photoplethysmograph (IR-PP) was made using an infrared-sensor (CNY70; wavelength 950 nm, Vishay, Malvern, PA, USA) and the circuit reported by Burnett (Burnett et al., 2013). The output of IR-PP was digitized and stored in a digital data recorder (MR8870, Hioki, Nagano, Japan) every 20 ms. In the original paper by Depledge (Depledge, 1984), the output voltage of the IR-PPG increased when reflected light increased. A typical heartbeat pattern (Fig. 4 in Depledge and Andersen, 1990) was presented with this polarity; however, in order to avoid counting error of the heart rate caused by double peaks, they put an inversion circuit in their plethysmograph. Burnett also employed this inversed polarity, and the output voltage (Vi) increased when reflected light decreased (Burnett et al., 2013). Therefore, we simply re-inversed the polarity by numerical calculation: 5-Vi, since the dynamic range of the circuit is around 5 volts. The IR-PP function was tested by an infrared (IR) pulse (1 s) driven by a block-pulse generator (Fig. S1A). We observed a slow decay, which might have been caused by transient response of the circuit and the phototransistor of CNY70. It is also true that the waveform obtained from living *Mytilus* was similar to that reported by *Mytilus edulis* (Bakhmet et al., 2009), even though the polarity is inversed. For the MRI study, a non-magnetic fiber optic probe was used for photoplethysmography (POXS-D; fiber bundle diameter: 3 mm; wavelength 940 nm, SA Instruments Inc., New York, USA). The emission of IR light and the detection of reflexed light were controlled by a small animal monitoring system (1025L, SA Instruments Inc., New York, USA). Then, the reflected light intensity was transferred, digitized and stored in a digital data recorder (MR8870, Hioki, Nagano, Japan) every 20 ms. The response of this system was faster than that of the original IR-PP with CNY70 (Fig. S1B), since the 1025L was optimized for the higher heart rate of rats and mice [300-600 beats per min (bpm)].

### Magnetic resonance imaging

The motion of the heart and the flow of the hemolymph were imaged by the inflow effect of *T*_1w_-MRI (Bock et al., 2001; Seo et al., 2014b). The MRI of *Mytilus galloprovincialis* in this study used procedures noted in a previous report (Seo et al., 2014a). In brief, a fiber optic probe (POXS-D) was fixed to the shell of the mussel by adhesive rubber (Blu-tack, Bostick, Victoria, Australia), and placed in a plastic tube (inner diameter of 22.5 mm). The mussels were emersed or immersed in 7 ml of synthetic seawater without aeration or circulation of seawater. The *Mytilus* has a metabolic system adapted to anaerobic conditions (de Zwaan and Wijsman, 1976). Indeed, the heart rate of *M. californianus* and the oxygen tension of the fluid in the mantle cavity stay almost constant during 1 to 6 h after the start of aerial exposure (Bayne et al., 1976). Therefore, the measurements were usually finished within 6 h. In order to change the heart rate, we considered measurements after 6 h as an anaerobic condition (hypoxia). In order to get a wide range of heart rates, in addition to aerial exposure and hypoxia, temperature was rapidly reduced from 21°C to 10°C within 1.5 h by using variable temperature control units (ECU-20 and BVT-2000, Bruker Biospin, Baden-Württemberg, Germany). The ^1^H MRI images were obtained by a 7T MRI system (AVANCE III, Bruker Biospin, Baden-Württemberg, Germany) with ParaVison operating software (version 5.1). A 7T vertical-bore magnet with a free bore size of 89 mm was equipped with an active shielded gradient (micro2.5) and a 25-mm ^1^H birdcage radiofrequency coil specially designed for 3% NaCl solution. The typical sagittal imaging parameters used for the *in vivo T*_1w_-MRI were as follows: 48×24 mm field of view (FOV) with a 128×64 data matrix and a slice thickness of 1 or 2 mm, 10 ms relaxation delay (*T*_R_), 3.5 ms echo-time (*T*_E_), 22.5° flip angle (*FA*), and 1 accumulation. A transverse image was obtained by 24×24 mm FOV with a slice thickness of 1 mm. Each MRI measurement session consisted of 128 images obtained every 0.64 s, and a 15-min interval was taken between the MRI sessions. For higher heart rates, the data matrix size was reduced to 64×32, the flip angle was adjusted to 11.3° or 15.9°, and 128 or 256 images were obtained every 0.32 s.

In separate experiments, in order to test the stability of the temperature of the sample, the temperature of the seawater (10 ml) was measured by a fluorescence thermometer (AMOS FX-8000-210, Anritsu Meter, Tokyo, Japan) before, during and after the MRI session at 30 s intervals. As shown in Fig. S2, we did not detect any temperature increase of more than 0.2°C in the four MRI experimental conditions: (1) 256 images with 0.64 s interval with *T_R_/T_E_/FA*=10 ms/3.5 ms/22.5°, and (2) 256 images with 0.32 s interval with *T_R_/T_E_/FA*=10 ms/3.5 ms/15.9° at 20°C or 10°C. We also did not detect any convection flow of the seawater during the MRI measurements. Therefore, we concluded that direct heating due to radio-frequency pulse is negligible in these experimental conditions.
